# Tropospheric attenuation prediction for future millimeter wave terrestrial systems: Estimating statistics and extremes

**DOI:** 10.1002/dac.5240

**Published:** 2022-05-30

**Authors:** Jinwen Liu, David W. Matolak, Ismail Güvenç, Hani Mehrpouyan

**Affiliations:** ^1^ Department of Electrical Engineering University of South Carolina Columbia South Carolina USA; ^2^ Department of Electrical and Computer Engineering NC State University Raleigh North Carolina USA; ^3^ Electrical and Computer Engineering Boise State University Boise Idaho USA

**Keywords:** tropospheric attenuation, worst month variability

## Abstract

Tropospheric attenuations can be significant in the millimeter wave (mmWave) frequency bands; hence, accurate prediction modeling of tropospheric attenuation is important for reliable mmWave communication. Several models have been established by the International Telecommunication Union (ITU), yet estimation accuracy is limited due to the large spatial scales used for model input parameters. In this paper, we address this and apply local precipitation data to analyze tropospheric attenuation statistics and compare to results when using ITU regional input rain data. Specifically, tropospheric attenuation is predicted via simulations using the ITU method at 30, 60, and 90 GHz in four distinct geographic locations with different climate types. From our simulations, we gather statistics for annual average rain attenuation, worst month rain attenuation, and rain attenuation per decade. Our results indicate that when using local measured rain data, for 1 km link distance, mean rain event attenuation increases from 0.5 to 2 dB. Local rain data yield larger attenuations at essentially all percentages of time not exceeded (essentially corresponding to all probability values): for example, for 0.1% of time not exceeded, in Columbia, SC, rain attenuation for 30 GHz frequency increases to 9 dB with local rain data, compared to 5 dB with ITU's regional data, corresponding to rain rates of 38.2 and 17.5 mm/h, respectively; at the same probability and location, the 90 GHz attenuation increases by 10 dB, from 10 to 20 dB when local rain data are used. Fog attenuations are also appreciable, reaching 8 dB for the 90 GHz frequency. Moreover, for the example locations, peak rain attenuations have increased at a rate of approximately 2 dB/decade over the past 50 years. Our results indicate that actual tropospheric attenuations may be substantially larger than that predicted by the ITU model when using regional rain rate data.

## Graphical abstract

In this paper, we address this and apply local precipitation data to analyze tropospheric attenuation statistics and compare to results when using ITU regional input rain data. Specifically, tropospheric attenuation is predicted via simulations using the ITU method at 30, 60, and 90 GHz in four distinct geographic locations with different climate types. Our results indicate that when using local measured rain data, for 1 km link distance, mean rain event attenuation increases from 0.5 to 2 dB.

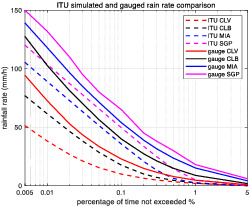

## 1 INTRODUCTION

Next generation communication systems are looking to the millimeter wave (mmWave) bands for the very wide bandwidths they can provide. According to Gopal,^1^ the peak download rate in 5G technology is designed to reach 5.8–10 GB/s. Since high‐rate communication will be in demand, higher frequency bands such as the mmWave bands will be required to provide sufficient bandwidths to support these rates. Yet electromagnetic waves suffer much larger attenuation in the mmWave bands (30–300 GHz) than in microwave bands; in the former, tropospheric attenuation can be a significant component.^2^

Tropospheric attenuation due to hydrometeors, clouds, and atmospheric gases can be large in these bands, and hence this may require large and expensive link margins or undesirably short link distances. For example, rain, which is typically the most significant hydrometeor in terms of attenuation, can cause attenuations of several tens of dB during storms. Accurate quantitative modeling for tropospheric attenuation is thus essential to guarantee reliable mmWave communication. Apart from annual average attenuation modeling, variability in extreme conditions, peak attenuations, and multi‐year variation must be quantified to accurately estimate link budgets for reliable mmWave links.

The International Telecommunication Union (ITU) is the worldwide source for best practices in much of radio engineering. ITU's standards for tropospheric attenuation are both theoretically based and empirical. The standard of interest here is ITU,^3^ which provides a detailed method for creating time series simulations of tropospheric attenuations using ITU's empirical formulas from a set of ITU‐R recommendations.^4^, ^5^, ^6^, ^7^, ^8^, ^9^ This simulation model requires inputs that are specific to geographic locations.

The empirical models and the simulation inputs are based on thousands of hours of measured data all over the world. Yet the inputs (especially rain rate) used for time series simulations may not be accurate enough for specific local areas. This is the case when measured data may come from stations that are significantly distant from the location of interest (e.g., several hundred km). This can also be the case when notable climate changes occur over a number of years at a given location. Thus, it is of interest to compare the accuracy of tropospheric attenuations with these two different types of inputs. This is the focus of this paper, where we compare results of the ITU tropospheric attenuation simulator when using two distinct inputs: regional ITU rain data and local measured rain data.

We apply measured precipitation data from the US National Oceanic and Atmospheric Administration (NOAA) online climate database^10^ to analyze local rain attenuation in three different climate regions of the United States: Cleveland, OH (humid continental climate), Columbia, SC (humid subtropical climate), and Miami, FL (tropical savanna climate). Rain rate data from Singapore^11^ are also used to analyze a typical location in a tropical rainforest climate region. The region classifications are according to the Köppen climate classification system. Frequencies of 30, 60, and 90 GHz were selected to represent communication frequencies spanning a range of the mmWave band, corresponding to Ka, V, and W bands, respectively. These local measured data sets are used as one set of inputs to the ITU tropospheric attenuation simulator. The second set of simulator inputs is regional rain data provided by the ITU. Outputs from simulations with the two different input data sets are then compared.

Note that NOAA's data were gathered in urban airports, where terrain is relatively flat. In addition, micro‐climate changes are not considered in this paper, since these locations could be regarded as typical climate regions. NOAA rain rate data were collected and integrated every 1 h during this 20‐year period, for three US locations. In contrast, rain rate data in Singapore were collected every 1 min. Hence, we converted all rain rate data to 1 min integration data, using the EXCELL‐RSC model described in ITU and Emiliani et al.^12^, ^13^ This method provides the smallest value of error compared to other empirical models for 1 h to 1 min integration time conversion. Comparing to alternative semi‐empirical method of Lavergnat and Gole (LG), EXCELL‐RSC method reduces RMS of error by 4%, approximately.

The subsequent sections are organized as follows. Section 2 provides a concise review of some related literature. In Section 3, the tropospheric attenuation models and the two different types of rain data inputs are described. Section 4 provides example time series results for each attenuation component (rain, gaseous, etc.). In Section 5, we discuss the worst month attenuation variability, of use to analyze link budgets in extreme rainy conditions. Attenuation variability over five decades, specifically 1963–2012, is described in Section 6, to analyze long‐term attenuation variation trends. Conclusions are provided in Section 7.

## 2 LITERATURE REVIEW

Much research has appeared on tropospheric attenuation modeling over several decades, for both terrestrial and earth‐space links. Hence, for brevity, we provide only some example citations. The ITU has established standards for each of the attenuation components and, in addition, has developed some worst month analyses.^3^, ^4^, ^5^, ^6^, ^7^, ^8^, ^9^, ^14^, ^15^ These standards are based on both empirical data and physical formulas.

We note that the empirical data used by ITU usually only covers a few locations in each geographic region, and this may not be sufficiently accurate. Moreover, empirical data need to be updated periodically to keep track of climatic changes or trends. Several papers have been published to improve ITU's recommendations for each attenuation component, often pertaining to specific locations. Some recent papers are discussed next.

Shrestha et al.^16^ discussed rain specific attenuation and a frequency scaling approach for Seoul, South Korea. In their Ku‐band earth‐space communication link, rain attenuation was measured for the satellite Koreasat 6 at a frequency of 12.25 GHz during years 2013–2015. Comparing measured and ITU's simulated rain attenuation for this frequency, the authors found an overestimation of up to 5 dB in the ITU recommendation for probability values between 0.001% and 0.05% (absolute probability values 10^−5^ to 5 × 10^−4^, i.e., for rare events). Hence, the authors modified the rain attenuation empirical formula for their Ku‐band results. By using the Ku‐band rain rates, the authors estimated the attenuation for Ka‐band (19.8 and 20.73 GHz) by the attenuation generation method in ITU.^5^

Shrestha et al.^17^ presented rain rate and attenuation measurement results for terrestrial links at 38 and 75 GHz. Comparing several existing models with their measured attenuation data, they found that ITU's model^4^ was the most accurate. Yet a 40% relative error with respect to the ITU model still existed for their rare event data, at probability values less than 0.01%.

Related to regional variation, the spatial variation of attenuation was considered for an 11.142 GHz link for the NSS‐6 satellite that was monitored in Kolkata in India.^18^ In this paper, diversity gain of an earth‐space link was measured and discussed. The authors divided the rainy season into three periods: pre‐monsoon, monsoon, and post‐monsoon periods. The measurement system could be considered a single‐input multiple‐output (SIMO) system, where rain attenuation with a single receiving site and with two sites was compared. In the 5 km link experiment, measured diversity gain was 0.24 dB larger than the ITU's results for monsoon conditions, 1.18 dB larger for pre‐monsoon, and 1.56 dB higher for the post‐monsoon period.

In Nandi and Nandi,^19^ rain rate and attenuations for a 30 GHz link between tropical and temperate regions were compared, for both terrestrial and earth‐space links. The authors chose Kolkata, India as the tropical simulation location and Spino d'Adda, Italy as the temperate location. The terrestrial attenuation estimation was based on the empirical method of ITU.^4^, ^9^ The physical synthetic storm technique was used to compute earth‐space path rain attenuation. A 7 dB difference between tropical and temperate location attenuations was found for the 200 m terrestrial link, and a 14 dB difference between these two locations was found for the earth‐space link, clearly illustrating the well‐known geographic dependence of tropospheric attenuations.

Lu et al.^20^ demonstrated a new rain attenuation prediction model for earth‐space links based on the physics of electromagnetic scattering. They found that ITU's existing rain attenuation prediction models^3^ varied monotonically at low elevation angles and low latitudes. Consequently, they introduced a rain rate adjustment factor to improve accuracy beyond that of the ITU model. Without sufficient measured attenuation data, they used ITU's analysis guideline^21^ to assess the performance of their proposed model. They found that using this guideline reduced the root‐mean‐square error between their new model and the existing ITU model attenuation results by 10%.

The cited papers are mainly focused on locations outside the United States and at lower frequencies than are planned for future mmWave applications. Little has been published regarding higher frequencies, and to our knowledge, nothing quantitative on comparing ITU simulator model outputs with two different types of rain input data (local vs. regional). We address these topics in subsequent sections.

## 3 TERRESTRIAL MMWAVE CHANNEL TROPOSPHERIC ATTENUATION MODELING

### 3.1 Tropospheric attenuation model introduction

**FIGURE 1:**
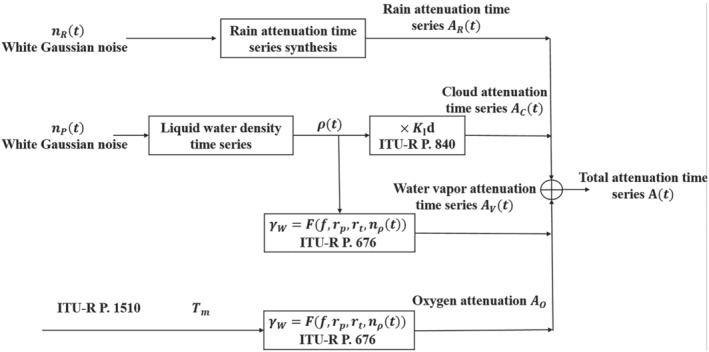
Tropospheric attenuation time series simulation block diagram

A block diagram of the ITU tropospheric attenuation time series simulation is shown in Figure 1. Attenuation consists of three components: rain attenuation, cloud attenuation, and gaseous attenuation, where gaseous attenuation can be further divided into oxygen attenuation and water vapor attenuation. Rain attenuation is derived from the rain rate, which is determined by location and temperature, via the ITU‐R method in ITU.^3^ Cloud attenuation^22^, * is an empirical function of liquid water density, visibility and temperature; this will be discussed in detail in Section 5. Water vapor attenuation is an empirical function determined solely by water vapor density, varying only by a relatively small amount for 10–100 GHz. Oxygen attenuation is an empirical function as well, determined by temperature. According to ITU,^4^, ^5^ rain and cloud attenuation time series follow a log‐normal distribution, whereas water vapor attenuation time series follow a Weibull distribution. However, for short path lengths in terrestrial channels, cloud and gaseous attenuation can in most cases be regarded as essentially constant for a specific link distance.^6^, ^7^, ^8^

### 3.2 Tropospheric attenuation time series generation

**FIGURE 2:**
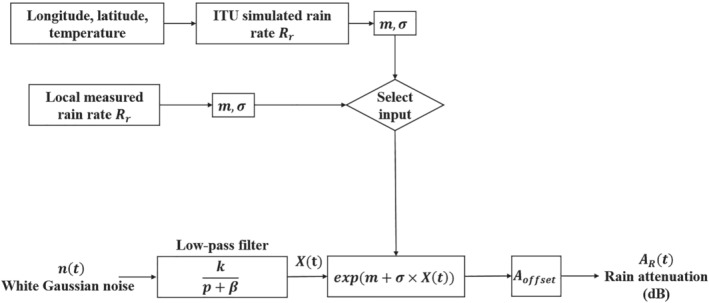
Rain attenuation time series synthesis block diagram

Rain attenuation time series are needed to simulate random rain attenuation over time. Since rain attenuation follows a log‐normal distribution, its time series can be created if the mean and standard deviation are known, along with the low pass filter used to provide proper temporal correlation. A least‐square fit between rain probability and rain rate is required for generating the mean (*m*) and standard deviation (*σ*) for the attenuation time series at any given location.^23^ For our rain attenuation time series, as illustrated in Figure 2, the log‐normal random process generation starts with a white Gaussian random process input to a low‐pass filter. Subsequently, a memoryless nonlinear device (exponentiation) is used, which is then followed by a calibration stage. The variable *p* in the filter block is the probability of rain, which ranges from 0.005% to 5% in this paper (a typical range, e.g., ITU^3^). Parameter *β* sets the bandwidth of the low‐pass filter, set to be 2 × 10^−4^ Hz as per ITU.^3^ The calibration parameter *A*_*offset*_ is defined in 3. This parameter primarily prevents the assumed rain probability of interest from exceeding the actual measurement‐based annual rain probability for the selected geographic location. The offset parameter is given by

(1) $$A_{\text{offset}}=e^{m+\sigma Q^{-1}\left(\frac{P_{0}}{100}\right)},$$

where *P*_*0*_ is the actual annual rain probability of the location of interest, which can be obtained from ITU,^23^ and *Q* ^−1^ is the inverse of the Gaussian “Q‐function,” defined as

(2) $$\begin{array}{c} Q\left(x\right)=\frac{1}{\sqrt{2\pi }}\int_{x}^{\infty }e^{-\frac{t^{2}}{2}}\mathrm{dt}. \end{array}$$

As indicated in Figure 1, the other components of tropospheric attenuation are cloud attenuation, oxygen attenuation, and water vapor attenuation. Cloud attenuation in terrestrial channels is generated by a Rayleigh approximation for Mie scattering model. For terrestrial links, clouds can be classified into thick fog (visibility on the order of 50 m) or thin fog (visibility on the order of 300 m) according to ITU's recommendation.^6^ For clear sky conditions, cloud attenuation is essentially negligible. Cloud, water vapor, and oxygen attenuations are simulated using the procedures and data described in the ITU recommendations as noted in Figure 1.

The total tropospheric attenuation, in dB, is obtained by adding these various attenuation components, where subscripts *R*, *O*, *C*, and *V* denote rain, oxygen, cloud, and water vapor components, respectively.

### 3.3 ITU regional and rain gauge data inputs

The rain rate required for rain attenuation simulation can be obtained from ITU's recommendation,^8^ by applying interpolation that is based on numerous sources of global rain data from The European Centre for Medium‐Range Weather Forecasts (ECMWF). Moreover, ITU suggests that simulations can use local data as input data sources, if local rain gauge data with detailed statistics are available. This process was followed in this paper.

As noted, four cities were selected to represent four climate regions: Cleveland, OH, as a temperate continental climate; Columbia, SC, as a subtropical humid climate; Miami, FL as a tropical humid climate; and Singapore, as a tropical rainforest climate. For Cleveland, Columbia, and Miami, we accessed the NOAA database for measured rain rate data.^10^ Measured rain rate data for Singapore was obtained from Leong and Chiann Foo.^11^ Corresponding to the dates of our rain gauge data, the period of 1985–2004 was selected for our simulations. This period was selected since the final model in ITU's recommendation^9^ was published in 2005. Hence, the time period was chosen for agreement among ITU's recommendation and the rain rate databases. The parameters of the simulations for these locations are listed in Table 1.

**TABLE 1:** Simulation parameters

	Location			
	Cleveland	Columbia	Miami	Singapore
Longitude	81.7 W	81.0 W	80.2 W	104.0 E
Latitude	41.5 N	34.0 N	25.8 N	1.4 N
Annual average temperature (°C)	6‐15	9‐22	21‐29	24‐32
Temperature in Simulation (°C)	9.9	16.5	23.8	26.4
Elevation above MSL (m)	210.3	91.4	3.7	15

## 4 TROPOSPHERIC ATTENUATION COMPARISON

### 4.1 Rain attenuation CCDF

In this paper, we consider a link length of 1 km for illustration, but all attenuations are essentially scalable with distance. Accordingly, the terrestrial link could be a typical point‐to‐point/multipoint link or even a low‐altitude aircraft link such as with an unmanned aerial vehicle (UAV) flying stably at low altitude, typically no more than 1 km above ground. For the simulation, we use a sampling frequency of 1 Hz and total simulation duration of 10^6^ s for a single simulation run, corresponding to approximately 11.5 days. Vertical polarization is assumed for the terrestrial link. These are all typical conditions for such tropospheric attenuation simulations.^18^

**FIGURE 3:**
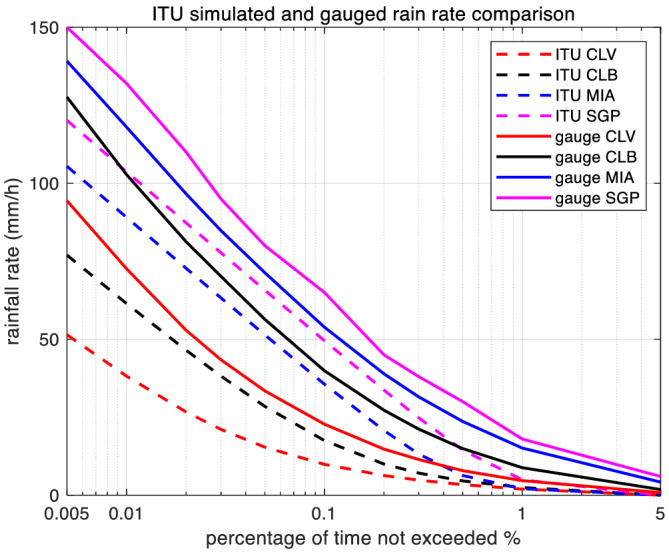
Annual average rain rate CCDF for four locations from two different data sources: ITU regional data and local measured rain rate

Figure 3 shows the resulting complementary cumulative distribution function (CCDF) curves^†^ for each of the four locations, for the period from 1985 to 2004, derived from two sources: ITU regional data for annual rain rate and actual measured rain rate. For all locations—our three US locations and Singapore—measured rain rate stays above the ITU regional data for all probabilities.^3^ At *p* = 0.1%, measured rain rate is approximately 15 mm/h larger than ITU's regional data.

Rain attenuation itself is determined by the specific rain attenuation $\gamma_{R}$ (dB/km) and the effective distance according to ITU^4^, ^6^

(3) $$\begin{array}{c} A_{R}=\gamma_{R}d_{\mathrm{eff}}. \end{array}$$

Since mmWave link distances for terrestrial links are expected to be short, potentially not exceeding 1 km, according to ITU,^4^ the value of *d*_*eff*_ is the actual link distance *d*. Specific rain attenuation γ in 4 is determined by^9^

(4) $$\begin{array}{c} \gamma_{R}=kR_{r}^{\alpha }. \end{array}$$

In 7, *k* and *α* are coefficients solely determined by frequency and polarization; note that α here is different with the parameter in 1, and $R_{r}$ is the rain rate in mm/h as in Figure 3. From curves in Figure 3, with the given assumption of log‐normality for rain attenuation, we can obtain the *m* and *σ* values for the lognormal random process generator for rain attenuation in Figure 2, by performing a least‐square fit, as in ITU.^9^

**FIGURE 4:**
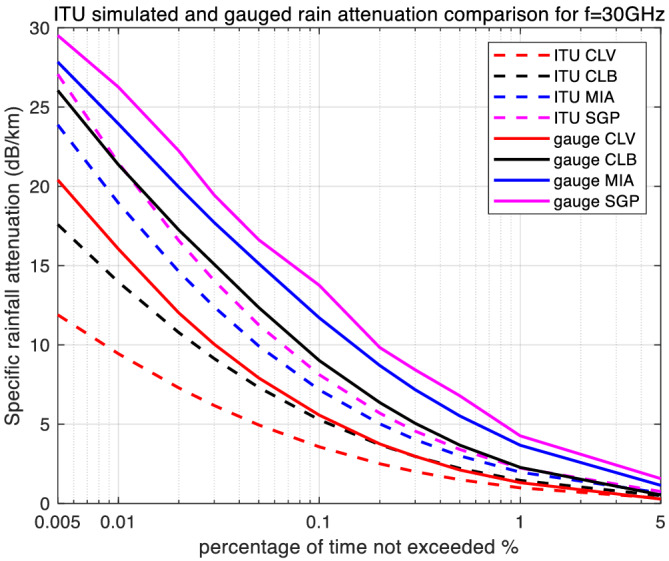
Simulated rain attenuation CCDF based on ITU's regional and local measured rain rate input, among four locations, for 30 GHz link

**FIGURE 5:**
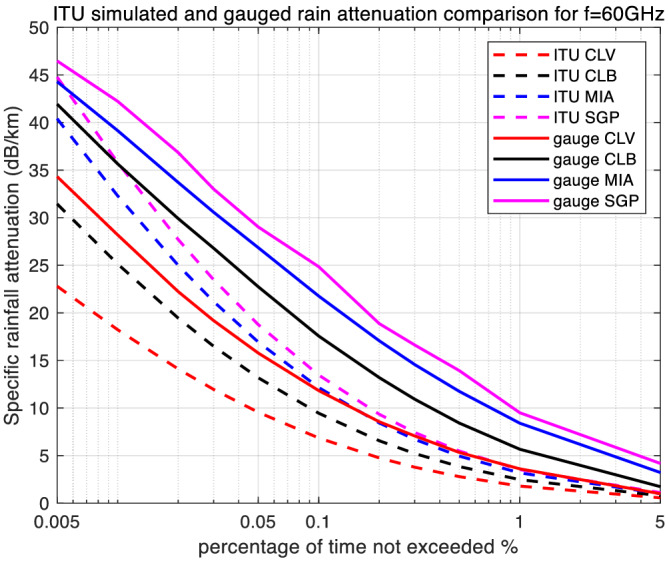
Simulated rain attenuation CCDF based on ITU's regional and local measured rain rate input, among four locations, for 60 GHz link

**FIGURE 6:**
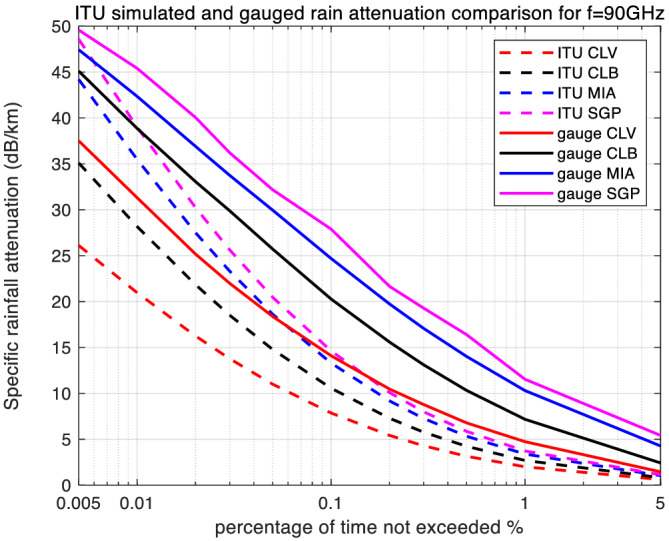
Simulated rain attenuation CCDF based on ITU's regional and local measured rain rate input, among four locations, for 90 GHz link

With the required lognormal parameters, we then generate simulation results for rain attenuation using both types of rain rate input sources. From a single simulation run of one million samples, rain attenuation CCDFs were generated for each of the four locations. These results are plotted for the three frequencies in Figures 4, 5, 6.

Rain attenuation CCDFs show the same shape and trend as the rain rate curves. For all three frequencies, and all four locations, the attenuation based on measured rain rate is larger than that based on the ITU regional data. Attenuation values also increase with frequency. For an example, at a probability of 0.1%, measured rain attenuation at 90 GHz is 2.96 dB larger than that at 60 GHz, and 13.92 dB larger than that at 30 GHz in Miami, FL and Singapore, as seen in Figures 4, 5, 6. In contrast, for the same probability value of 0.1%, using regional ITU input data, the Miami and Singapore attenuation at 90 GHz is only 1.15 dB larger than that at 60 GHz and only 6.15 dB larger than that at 30 GHz, illustrating relative differences across frequency when using the two different input data sets.

### 4.2 Rain attenuation time series comparison

**FIGURE 7:**
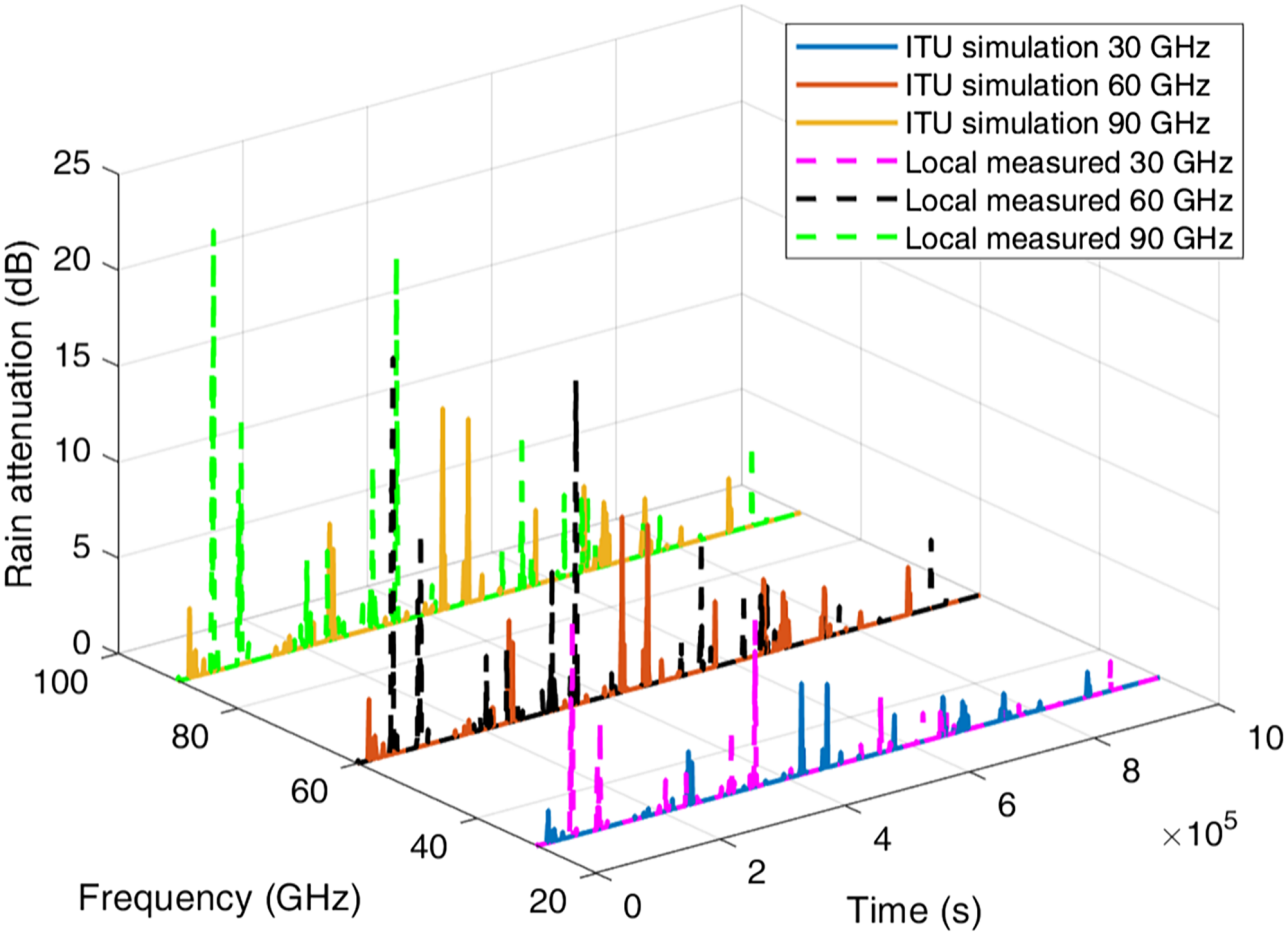
Rain attenuation time series for three mmWave frequencies based on ITU regional and local measured rain data input, for Cleveland, OH

**FIGURE 8:**
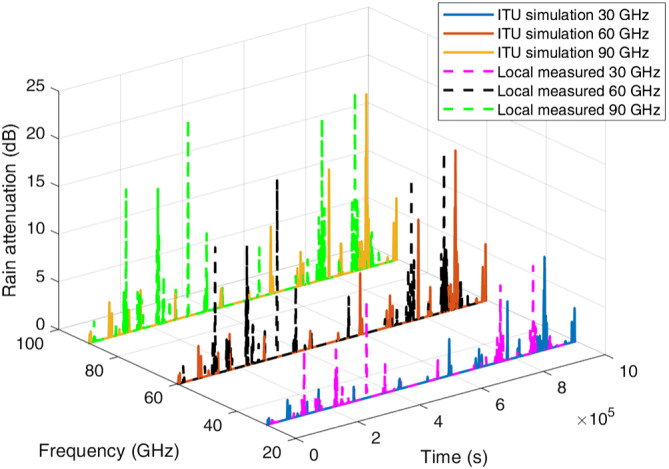
Rain attenuation time series for three mmWave frequencies based on ITU regional and local measured rain data input, in Columbia, SC

**FIGURE 9:**
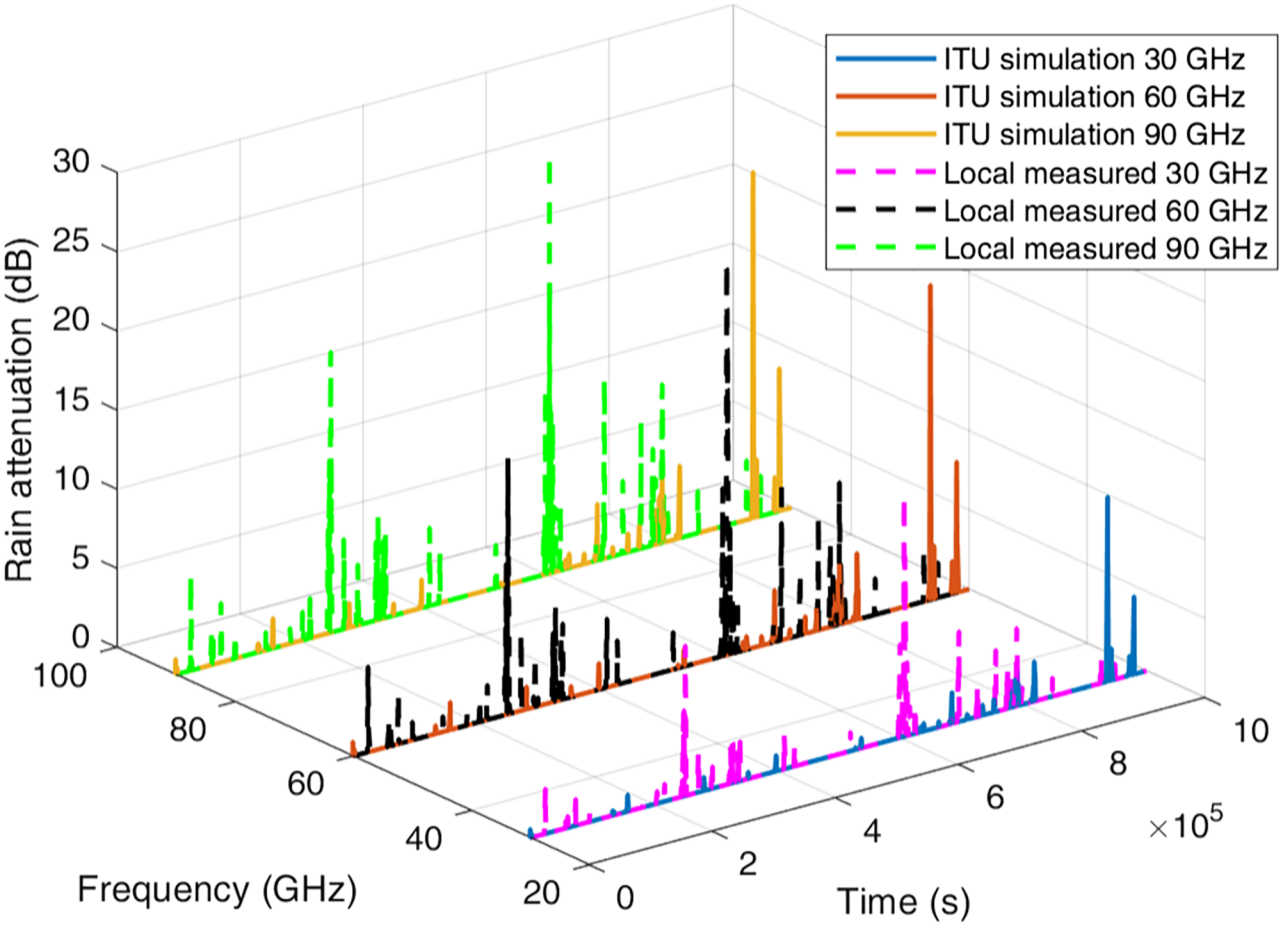
Rain attenuation time series for three mmWave frequencies based on ITU regional and local measured rain data input, for Miami, FL

**FIGURE 10:**
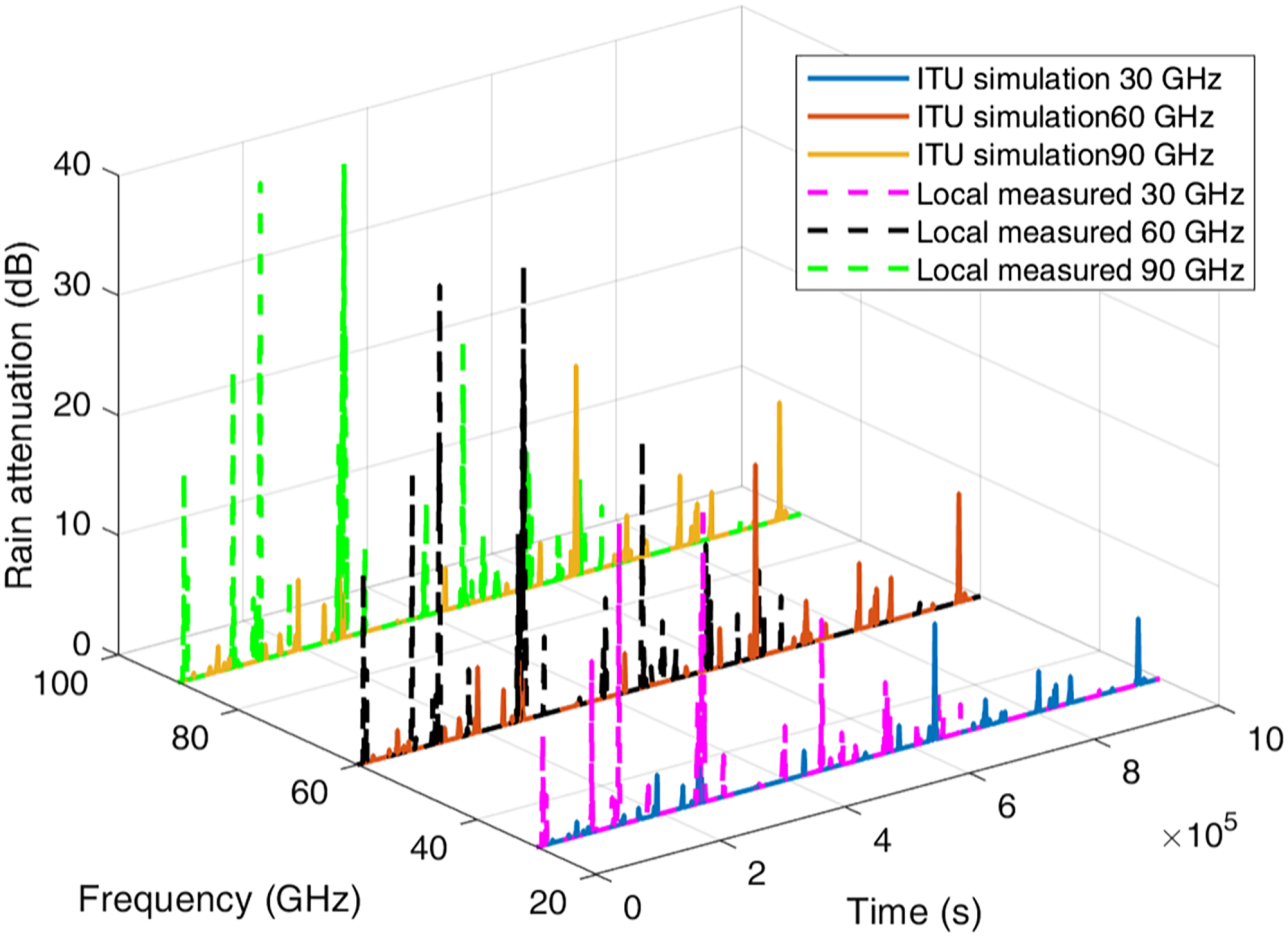
Rain attenuation time series for three mmWave frequencies based on ITU regional and local measured rain data input, for Singapore

Example‐simulated attenuation time series for the four locations at frequencies of 30, 60, and 90 GHz are plotted in Figures 7, 8, 9, 10. Note that although frequencies are different, the attenuation time series are generated from the same random input. That is, attenuations shown for each frequency are due to the exact same simulated rain events, for a given rain input data type (local measured or ITU regional). Since the simulation runs employ different inputs (local or ITU regional), different rain events occur when the input data are different; thus, we compare the effect of the different input data types via statistics, not via individual time series as shown in Figures 7, 8, 9, 10. It can be observed from Figures 7, 8, 9, 10 that for the US locations, there are a larger number of rain events when inputs are based on measured data, than when based on the ITU regional data. Statistical results from these simulations are listed in Table 2, averaged over 10 simulation runs (each of one million time samples). For all statistics, we count attenuations only during rain events, i.e., when rain rate is greater than zero.

**TABLE 2:** Simulated rain attenuations for ITU and local measured inputs, for 30, 60, and 90 GHz, 1 km link

Frequency	30 GHz		60 GHz		90 GHz	
	ITU	Local measured	ITU	Local measured	ITU	Local measured
Cleveland, OH						
Mean (dB)	0.47	0.89	0.88	2.00	1.00	2.42
Standard deviation (dB)	0.58	1.34	1.12	2.70	1.27	3.14
Peak (dB)	4.74	11.50	9.13	20.65	10.48	22.98
Heavy rain events (times)	1,018	5,102	563	4,553	458	4,399
Columbia, SC						
Mean (dB)	0.65	1.48	1.06	2.92	1.15	3.38
Standard deviation (dB)	0.59	1.46	1.04	3.74	1.15	4.23
Peak (dB)	8.16	16.07	12.39	26.54	18.22	28.79
Heavy rain events (times)	2,745	8,269	1,254	6,872	1,050	6,249
Miami, FL						
Mean (dB)	1.25	2.06	1.85	4.03	2.10	4.33
Standard deviation (dB)	1.62	2.33	2.75	3.96	3.01	4.31
Peak (dB)	11.34	18.25	19.31	28.87	20.99	30.60
Heavy rain events (times)	3,885	11,419	1818	9,854	1,510	9,495
Singapore						
Mean (dB)	1.31	2.28	2.13	4.10	2.43	4.55
Standard deviation (dB)	2.12	3.46	3.53	5.70	3.85	6.14
Peak (dB)	13.58	23.75	22.65	36.36	24.68	38.10
Heavy rain events (times)	4,931	15,031	2,559	12,180	2,290	11,640

We observe from Table 2 that the higher frequencies yield larger mean attenuation and also larger variability (as quantified by rain attenuation standard deviation), for both types of inputs. Attenuation based on measured rain rate has *larger* mean values and standard deviations than the attenuation based upon ITU's regional rain data, for all locations and frequencies. In terms of maximum (peak) attenuation, Singapore has the largest peak rain attenuation values among all locations, for all frequencies, whereas Cleveland has the smallest peak rain attenuation values.

Over all locations, the use of local measured data instead of ITU regional data increases the mean attenuation by approximately 0.5 dB at 30 GHz in Cleveland, OH and Columbia, SC, and it increases by 1 dB in Miami, FL and Singapore, for 30 GHz. For 60 and 90 GHz, the mean increases by approximately 1.5 dB in Cleveland, OH and Columbia, SC, compared to an increase of 2 dB in Miami, FL and Singapore. Regarding standard deviation, a 2 dB increase occurs using local measured data for Singapore, whereas the standard deviation increases by up to 1 dB in the three US locations, again over a 1 km link distance.

In terms of peak attenuation values, local measured data yields results that are larger than results based on ITU simulation in all four locations, in agreement with curves in Figures 3, 4, 5, 6. In addition, as noted, more “heavy rain events” occur using local measured input than when using ITU's regional input. Heavy rain is defined as a rainfall rate larger than 7.5 mm/h,^24^ corresponding to 2.52, 6.16, and 7.29 dB rain attenuation values for 30, 60, and 90 GHz 1 km links, respectively. The number of heavy rain event table entries in Table 2 denote the number of samples out of 10 simulation runs (10^7^ total samples) that exceed the rainfall rate value 7.5 mm/h. Thus, rain attenuation time series based on local measured rain rate input have more frequent heavy rain events than when based on ITU's input.

### 4.3 Cloud and gaseous attenuation

**FIGURE 11:**
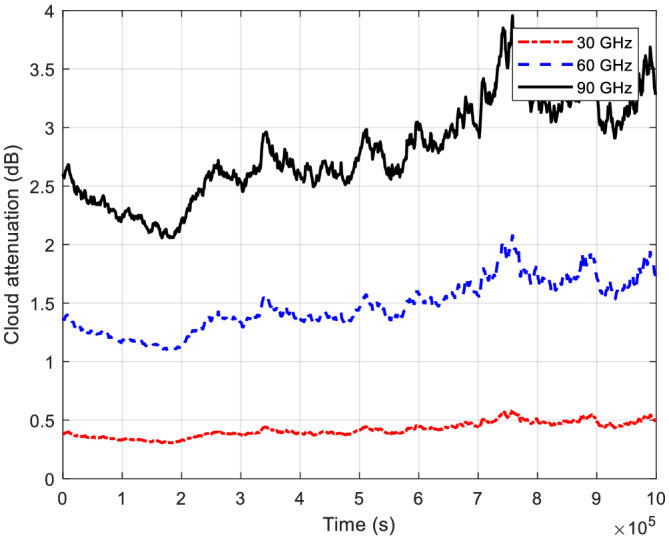
Cloud attenuation time series for three frequencies in Cleveland, OH, for all three frequencies, 1 km link distance

Although rain attenuation is much larger than that of the other tropospheric attenuation components, quantification of the other attenuation components is still important. Figure 11 shows example simulated cloud attenuation time series for Cleveland, OH. Cloud attenuation statistics from simulations for other locations are listed in Table 3. Cloud attenuation increases with frequency and also tends to increase with colder temperatures. Hence, of our four example locations, Cleveland, OH incurs the largest cloud attenuation. These values correspond to heavy fog conditions for terrestrial links. For light fog conditions, the attenuations are approximately one tenth of the values in Table 3. For clear sky conditions, cloud/fog attenuation can be neglected.

**TABLE 3:** Simulated cloud and gaseous attenuations, in dB for ITU inputs, for 30, 60, and 90 GHz, at four locations, 1 km link

Frequency	30 GHz		60 GHz		90 GHz	
	Cloud	Gas	Cloud	Gas	Cloud	Gas
Cleveland, OH						
Mean	0.56	0.03	2.00	15.71	3.79	0.06
Standard deviation	0.15	0	0.52	0	0.98	0
Peak	1.64	0.03	5.88	15.71	11.15	0.06
Columbia, SC						
Mean	0.41	0.03	1.50	14.82	2.96	0.06
Standard deviation	0.05	0	0.20	0	0.39	0
Peak	0.74	0.03	2.67	14.82	5.61	0.06
Miami, FL						
Mean	0.33	0.03	1.24	13.92	2.54	0.06
Standard deviation	0.01	0	0.04	0	0.09	0
Peak	0.39	0.03	1.47	13.92	3.03	0.06
Singapore						
Mean	0.31	0.03	1.17	13.60	2.42	0.06
Standard deviation	0.01	0	0.03	0	0.05	0
Peak	0.33	0.03	1.30	13.60	2.67	0.06

**FIGURE 12:**
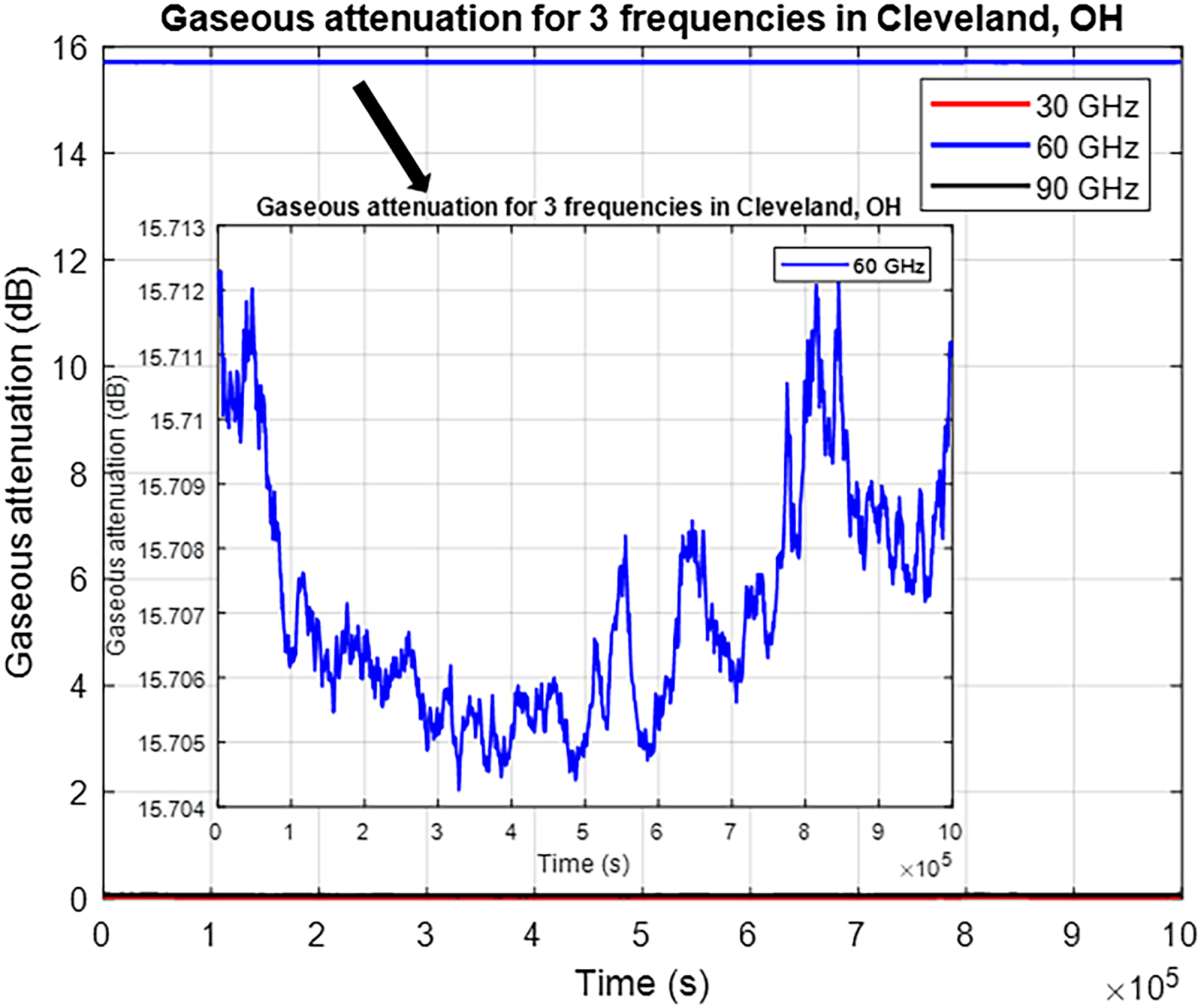
Gaseous attenuation time series for three frequencies in Cleveland, OH. Inset shows 60 GHz attenuation time series on expanded scale. Link distance is 1 km

Gaseous attenuation can be divided into oxygen and water vapor attenuation. Both components scale with distance according to ITU.^7^ Water vapor attenuation is very small and is on the order of 0.001 dB. For a specific frequency, oxygen attenuation is constant for a constant temperature and air pressure (9.9°C for Cleveland as listed in Table 1). Figure 12 shows gaseous attenuation in Cleveland versus time for a 1 km link distance. We note that oxygen attenuation is fairly large at 16 dB for the 60 GHz link. This is a special phenomenon caused by oxygen absorption. The oxygen molecule ( $O_{2}$) absorbs electromagnetic energy at 60 GHz. This phenomenon is described in ITU‐R P.676 in detail.

### 4.4 Total tropospheric attenuation comparison

Total tropospheric attenuation is obtained by adding attenuations of the various components, as in 5. Statistics for rain, cloud, and gaseous attenuations from our simulations are listed in Tables 2 and 3. Rain and cloud attenuation increase with frequency, whereas oxygen attenuation is maximum at 60 GHz. Hence, total tropospheric attenuation for 60 GHz links is generally largest. For example, for a 1 km link, mean attenuation at 60 GHz is approximately 18 and 14 dB larger than at 30 and 90 GHz, respectively. If we include the free space path loss, the attenuation of the 60 GHz link is still largest, at 24 and 10.5 dB larger than that of the 30 and 90 GHz frequencies, respectively.

The different locations are of different climate types, encompassing differences in temperature, rain rate, and humidity. Among the four locations chosen in this paper, rain attenuation ranks from largest to smallest in Singapore (rainforest climate), then Miami (tropical humid climate), then Columbia (subtropical humid climate), then Cleveland (temperate continental climate). In terms of peak attenuation values, rain attenuation based on local measured rain data in Singapore can be as much as 20 dB larger than that in Cleveland. Cloud and gaseous attenuation rank in the reverse manner, since cloud attenuation decreases with temperature. Thus, for the 60 GHz link example, mean cloud and gaseous attenuation in Cleveland, OH is approximately 1 and 2 dB larger than that for Singapore.

## 5 TROSPOSPHERIC ATTENUATION VARIABILITY

### 5.1 Worst month rain attenuation variablity

The worst month is defined in ITU^25^ as the month with the highest rain rate (as measured over some time period, usually several decades). As noted, since rain attenuation scales with rain rate, the worst months are those with the largest rain rates, and hence the largest rain attenuations. For communication link reliability, worst month variability is of prime importance. Since rain attenuation variability dominates tropospheric attenuation variability, worst month rain attenuation variability is sufficient to analyze total variability. In ITU's recommendation,^13^ the worst month rain attenuation is derived by applying 8:

(5) $$\begin{array}{c} q=\frac{p_{w}}{p}=\left\{\begin{array}{c} 12,\;\text{for}\;p<{\left(\frac{Q_{1}}{12}\right)}^{\frac{1}{\kappa }}\%\\ Q_{1}p^{-\kappa },\;\text{for}\;{\left(\frac{Q_{1}}{12}\right)}^{\frac{1}{\kappa }}\%<p\leq 3\%\\ Q_{1}3^{-\kappa },\text{for}\;3\%<p\leq 30\% \end{array}\right.. \end{array}$$

The parameter *q* in 8 denotes the ratio between the worst month probability *p*_*w*_ and annual average probability *p* of exceeding a specific attenuation value. The parameter κ in 8 is an empirical constant. As an example, if in annual average conditions, rain attenuation not exceeding 10 dB occurs with a probability 0.05% (absolute probability 0.0005), this probability becomes 1.12% after conversion to the worst month attenuation value. Parameters *Q*_1_ and κ are coefficients depending on climate region. For global use, *Q*_1_ is 2.85, and κ is 0.13. Therefore, worst month rain attenuation based on ITU's regional input data can be obtained from annual average rain attenuation in Section 4.

In terms of rain attenuation based on measured rain rates, the worst month is simply defined by the month with largest measured rain rate. According to NOAA,^10^ the worst months in Columbia, SC, and Cleveland, OH, are July and August, whereas in Miami, FL, the worst months are August and September, based upon the period from 1985 to 2004. There are two “worst months” for these cities since the rain rate statistics for these two months are very nearly identical. As a result, when considering Columbia, SC, either July or August can be used as a representative worst month. Worst month rain attenuation must utilize monthly rain rate data instead of commonly used annual rain rate data, so we analyze only the three US locations for worst month variability.

**FIGURE 13:**
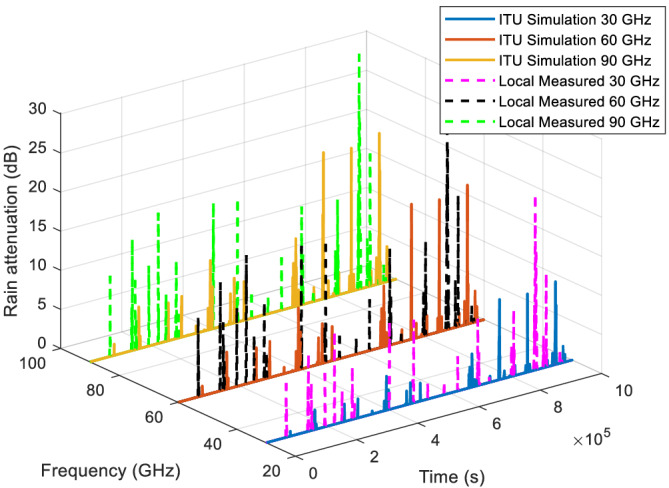
Simulated worst month rain attenuation time series based on both ITU's regional input data and local measured data for three frequencies in Miami, FL

Figure 13 shows example worst month rain attenuation time series for Miami, FL, for the two different types of input rain data. The mean, standard deviations and peaks of attenuation using measured input data are all again larger than those using ITU's regional input data. This agrees with the rain rate CCDFs of Figures 4, 5, 6.

Table 4 shows the statistics of worst month rain attenuation for these three locations at the three frequencies, averaged from another set of 10 simulation runs (different from Table 2). In most cases, mean attenuation increases by approximately 0.5–1 dB compared to values in Table 2, and standard deviation increases by 2 dB. Comparing the maxima, the peak attenuation exceeds that of the average annual values of Table 2 by 5–10 dB in the three locations and frequencies. The definition of heavy rain events is the same as in Table 2. Approximately 1,000–4,000 more events occur in these worst month cases, comparing to annual rain statistics in Table 2, which means that heavy rain occurs much more frequently during measured worst months. Future study of such events may be able to exploit modeling of statistical extremes.^26^

**TABLE 4:** Simulated worst month rain attenuations, in dB, for ITU and NOAA inputs, for 30, 60, and 90 GHZ, for four locations, 1 km link

Frequency	30 GHz		60 GHz		90 GHz	
	ITU	Local measured	ITU	Local measured	ITU	Local measured
Cleveland, OH						
Mean	0.93	2.03	1.79	3.37	2.05	3.63
Standard deviation	1.41	2.55	2.75	4.03	3.18	4.26
Peak	12.91	18.53	25.34	27.68	28.67	29.42
Heavy rain Events	2,350	9,055	1,637	3,617	1,567	2,401
Columbia, SC						
Mean	1.75	2.83	3.15	4.61	4.13	4.93
Standard deviation	2.62	2.89	4.51	4.74	4.73	4.99
Peak	17.35	21.86	26.89	30.57	28.81	32.09
Heavy rain events	3,623	9,867	2,682	4,682	2,244	3,043
Miami, FL						
Mean	2.78	3.66	3.29	5.32	4.67	5.45
Standard deviation	3.96	5.04	4.72	6.52	4.97	7.50
Peak	25.35	36.54	43.72	47.67	48.37	49.97
Heavy rain events	8,586	13,562	6,198	6,699	3,556	4,752

### 5.2 Cloud attenuation variablity

Cloud (or fog) attenuation *A*_*C*_ plotted in Figure 11 denotes the radiation fog case where visibility is 50 m. In Zhao and Wu and Maher et al.,^27^, ^28^ fog is divided into advection fog and radiation fog. The specific cloud attenuation value is a function of the liquid water content,^6^ which is further determined by fog type and visibility as in the following empirical formulas based on the Rayleigh scattering model^27^

(6) $$\begin{array}{c} A_{C}=\gamma_{c}d, \end{array}$$

(7) $$\begin{array}{c} \gamma_{c}=K_{l}M, \end{array}$$

(8) $$\begin{array}{c} M_{\mathrm{adv}}=0.0156\;V^{-1.43}, \end{array}$$

(9) $$\begin{array}{c} M_{\mathrm{rad}}=0.00316\;V^{-1.54}, \end{array}$$

where $\gamma_{c}$ is cloud specific attenuation in dB/km, *d* is link distance in km, *M* is liquid water density in g/m^3^, *V* is visibility in km, and $K_{l}$ is a constant dependent on frequency, temperature, and elevation angle, calculated in ITU.^3^

Table 5 lists additional fog attenuation simulation results in different visibility and fog type conditions for the worst case among our frequencies and locations, i.e., a 90 GHz link in Cleveland, OH. In the extreme case, advection fog (adv) may cause 33 dB more attenuation than radiation fog (rad) for the 30 m visibility case. For radiation fog, the peak attenuation value can increase by up to 8 dB if visibility decreases from 50 to 30 m.

**TABLE 5:** Simulated fog attenuation, in dB, under different visibility conditions in Cleveland, OH, 90 GHz, 1 km link

Visibility (m)	Mean		Standard deviation		Peak	
	adv	rad	adv	rad	adv	rad
30	27.94	8.32	7.22	2.15	47.85	14.25
50	13.46	3.79	3.48	0.98	23.05	6.49
100	5.00	1.30	1.29	0.34	8.55	2.23
200	1.85	0.45	0.48	0.12	3.17	0.77
300	1.04	0.24	0.27	0.06	1.78	0.41
500	0.50	0.11	0.13	0.03	0.86	0.19
1,000	0.19	0.04	0.05	0.01	0.32	0.06

Extreme attenuation analysis in this section provides a conservative estimate of tropospheric attenuation. Since rain attenuation and cloud attenuation based on measured data are at least several dB larger than simulation method described in ITU,^4^ link margin is necessary to be raised in terrestrial link communication. Even if results are based on random process, a more conservative consideration is still needed, especially for insurance of precise unmanned aircraft systems with high expense.

## 6 RAIN ATTENUATION PREDICTION

### 6.1 Rain rate trends

**FIGURE 14:**
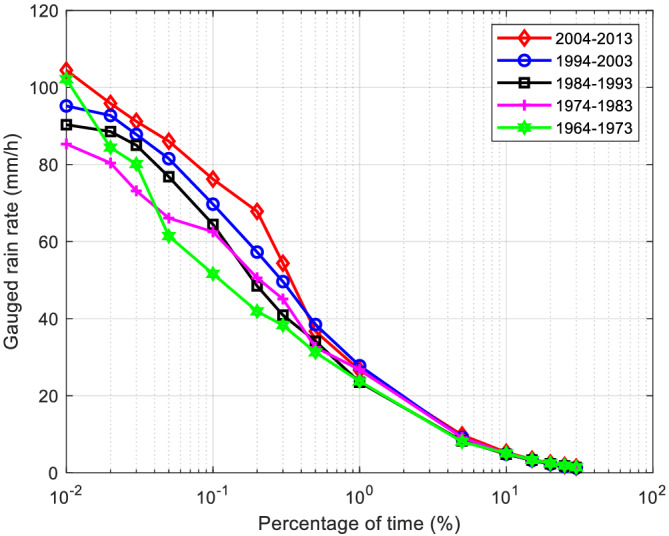
Measured rain rate CCDF for recent five decades in Columbia, SC

Due to measured global climate changes and El Nino effects, rain rates have substantially increased in many parts of the world in each decade during the years 1963–2013.^10^ The heaviest rainfall events have become heavier and more frequent decade after decade from 1963 to 2013 as well. Figure 14 shows the per‐decade measured rainfall rate for each decade from the years of 1963–2013 for Columbia, SC.

Table 6 shows the detailed per‐decade statistics of rainfall rate in Columbia, SC, derived from NOAA data.^10^ Overall the mean rain rate has increased by approximately 0.5 mm/h over this 50 year period. Heavy rain events and floods have occurred more often as well. Note that different from the heavy rain events in Tables 2 and 4, so‐called violent rain events here are derived from local measured rain data^10^ and have rain rates larger than 10 mm/h. These particular measurements are only sampled 3‐4 times a day, while rain data are converted to 1 minute integration. Note that same conversion method for rain data integration is used, given by Equations 1 and 2.

**TABLE 6:** Rain rate statistics (mm/h) for Columbia, SC across five decades

Decade	1964–1973	1974–1983	1984–1993	1994–2003	2004–2013
Mean	2.26	2.32	2.41	2.46	2.59
Standard deviation	3.90	3.69	4.40	4.75	4.38
Number of violent rain events (>10 mm/h)	196	213	216	231	245
Peak rain rate	102.20	85.32	90.32	95.20	104.50

### 6.2 Rain attenuation prediction

**FIGURE 15:**
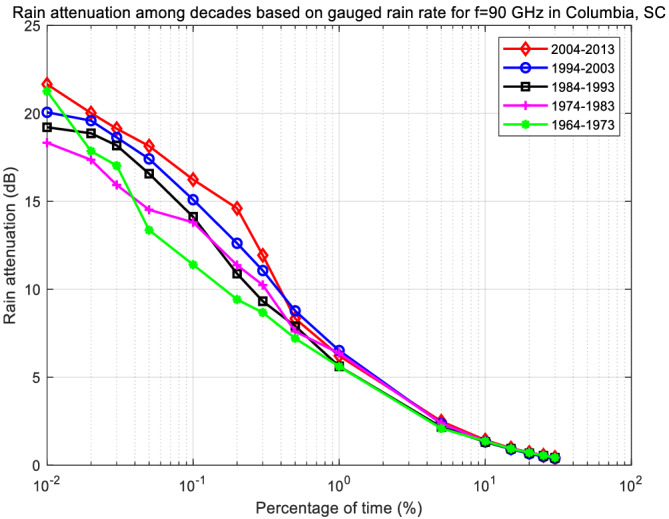
Rain attenuation CCDF based on local measured rain rate input for recent five decades in Columbia, SC, 30 GHz

**FIGURE 16:**
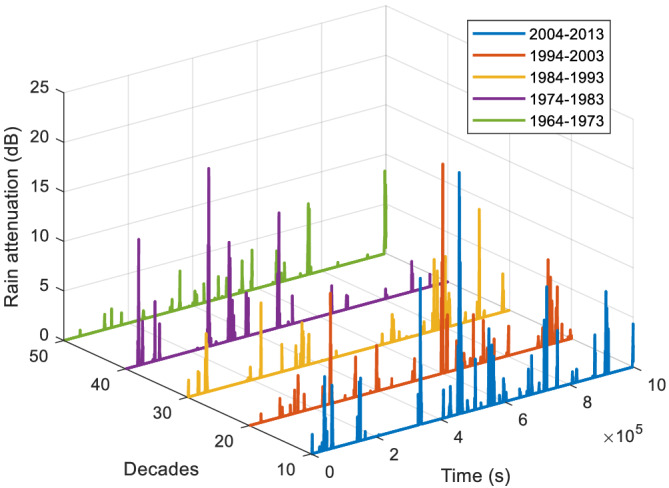
Rain attenuation time series based on local measured rain rate for recent five decades in Columbia, SC, 30 GHz

Based on the rain rate statistics in Table 6, attenuations for Columbia, SC, at 30 GHz were generated for each decade. Reference^29^ also shows some figures as evidence of increasing rain rate over recent decades. The resulting CCDFs are plotted in Figure 15. The relationship between rain rate and attenuation is that of 6 and 7. Following the same trend, attenuation values increase over the five‐decade period. A sample simulation run for each of the five decades is plotted in Figure 16. Note that attenuation time series in each decade are random and independent of each other. Table 7 lists the statistics of simulated rain attenuation for Columbia, SC, at our three frequencies, averaged from another 10 simulation runs (different from Tables 2 and 4). For 1 km link distance, an approximate 1 and 2 dB increase in attenuation mean and standard deviation, respectively, occur in the 50 year period from 1963 to 2013 for these frequencies and locations. For the peak values, an approximate 2 dB increase occurs per decade, comparing the decade of 2004–2013 to 1964–1973. Comparing with the 20 year period annual average attenuation in Figure 4, rain attenuation in decade 2004–2013 is 0.4 and 0.5 dB larger in the mean and the standard deviation, respectively.

**TABLE 7:** Statistics for rain attenuation in dB in Columbia, SC over five decades, for three frequencies

Decade	1964–1973	1974–1983	1984–1993	1994–2003	2004–2013
30 GHz					
Mean	0.74	0.86	0.94	1.18	1.58
Standard deviation	1.11	1.04	1.34	2.47	3.18
Peak	15.80	14.12	16.80	18.44	20.07
60 GHz					
Mean	1.33	1.46	1.77	2.18	2.49
Standard deviation	1.49	1.39	1.83	2.62	3.33
Peak	18.39	16.38	19.92	21.91	24.37
90 GHz					
Mean	1.68	1.89	2.30	2.64	2.94
Standard deviation	1.89	1.86	2.17	2.86	3.74
Peak	20.10	18.77	21.57	25.32	29.77

## 7 CONCLUSION

In this paper, we described tropospheric attenuation modeling for terrestrial links, for mmWave frequencies. The widely established ITU method was employed to assess the several attenuation components, including rain, clouds, and atmospheric gases oxygen and water vapor. The ITU method requires specific inputs tied to geographic locations, and since rain is the dominant attenuation component, we investigated the ITU model results with two different types of rain inputs: local measured rain data, and the ITU regional rain data. This was done for four specific locations spanning temperate to tropical climates (Cleveland, OH, Columbia, SC, Miami, FL, and Singapore) and for three mmWave frequencies, 30, 60, and 90 GHz. The use of local measured (rain) data yielded much more frequent rain events than the ITU regional data, and this yields an increase in mean and standard deviation of tropospheric attenuation at all locations and frequencies. Worst month attenuations were analyzed as well, and further increases were seen with local measured rain data in these months. Variability over a 50 year period was also analyzed in the US locations. Both annual average rain rate (and hence attenuation) and the number of heavy rain events have increased over this period. An approximate 2 dB increase in peak rain attenuation was found for each decade, again over 1 km link distance. These results taken together indicate that, (i) as ITU recommends, local measured input data should be used whenever possible to assess tropospheric attenuations, (ii) measured local rain data shows more frequent rain events in all our selected locations, and this contributes to larger mean and standard deviation attenuation values for all frequencies, and (iii) the number of rain events and rain intensities have increased over the past half‐century, which may portend even larger future rain rates and mmWave rain attenuations. Future work will investigate tropospheric attenuation variability and additional statistical modeling of extreme attenuation values.

## Data Availability

The data that support the findings of this study are openly available in [10] NOAA climate data online database at https://www.ncdc.noaa.gov/cdo-web/.

## ENDNOTES

* We note that when the cloud is within the planetary boundary layer, it is termed fog.^19^

^†^
Note these CCDFs are “rotated,” with time percentage the abscissa and attenuation the ordinate, rather than the reverse. Also, times are given in percentages instead of absolute probabilities. Both of these are standard ITU conventions.
